# Isolation and characterization of alternatively spliced variants of the mouse sigma_1_ receptor gene, Sigmar1

**DOI:** 10.1371/journal.pone.0174694

**Published:** 2017-03-28

**Authors:** Ling Pan, David A. Pasternak, Jin Xu, Mingming Xu, Zhigang Lu, Gavril W. Pasternak, Ying-Xian Pan

**Affiliations:** 1 Department of Neurology and the Molecular Pharmacology Program, Memorial Sloan-Kettering Cancer Center, New York, New York, United States of America; 2 Key Laboratory of Acupuncture and Medicine Research of Ministry of Education, Nanjing University of Chinese Medicine, Nanjing, Jianshu, China; 3 First Clinical Medical College, Nanjing University of Chinese Medicine, Nanjing, Jiangshu, China; National University of Singapore, SINGAPORE

## Abstract

The sigma_1_ receptor acts as a chaperone at the endoplasmic reticulum, associates with multiple proteins in various cellular systems, and involves in a number of diseases, such as addiction, pain, cancer and psychiatric disorders. The sigma_1_ receptor is encoded by the single copy SIGMAR1 gene. The current study identifies five alternatively spliced variants of the mouse sigma_1_ receptor gene using a polymerase chain reaction cloning approach. All the splice variants are generated by exon skipping or alternative 3’ or 5’ splicing, producing the truncated sigma_1_ receptor. Similar alternative splicing has been observed in the human SIGMAR1 gene based on the molecular cloning or genome sequence prediction, suggesting conservation of alternative splicing of SIGMAR1 gene. Using quantitative polymerase chain reactions, we demonstrate differential expression of several splice variants in mouse tissues and brain regions. When expressed in HEK293 cells, all the splice variants fail to bind sigma ligands, implicating that each truncated region in these splice variants is important for ligand binding. However, co-immunoprecipitation (Co-IP) study in HEK293 cells co-transfected with tagged constructs reveals that all the splice variants maintain their ability to physically associate with a mu opioid receptor (mMOR-1), providing useful information to correlate the motifs/sequences necessary for their physical association. Furthermore, a competition Co-IP study showed that all the variants can disrupt in a dose-dependent manner the dimerization of the original sigma_1_ receptor with mMOR-1, suggesting a potential dominant negative function and providing significant insights into their function.

## Introduction

Sigma receptors were initially proposed following the pharmacological studies with the benzomorphan opiate (±)SKF-10047 [1]. Although initially thought to be related to opioid receptors, the sigma receptor is now recognized as a distinct protein unrelated to any traditional classes of receptor, including opioid receptors [2, 3]. Two subtypes of sigma receptors, sigma_1_ and sigma_2_ receptors, have been proposed based on their binding selectivity profiles [4]. Sigma receptors are expressed in almost all tissues [5, 6] and in a wide range of tumors [7–9].

The sigma_1_ receptor was first cloned from guinea pig liver after purification and partial sequencing of the protein [10]. This quickly led to the identification of human [11], mouse [12, 13], and rat [14, 15] homologs by screening cDNA libraries or PCR cloning. The gene structure and chromosomal location of the sigma_1_ receptor gene, SIGMAR1, were soon identified in mouse and human [12, 13, 16]. So far, only a single copy of the SIGMAR1 gene has been described. The predicted amino acid sequences of the cloned sigma_1_ receptors share high homology (> 89% identity) among mouse, rat and human, but they are not structurally homologous with any of known mammalian proteins, except for a modest homology with fungal proteins involved in sterol synthesis [10]. All the cloned sigma_1_ receptors displayed similar binding profiles resembling the sigma_1_ binding site defined in early pharmacological studies [10–15].

The sigma_1_ receptor functions as a chaperon within the endoplasmic reticulum [17], associates with many of proteins in various cellular systems [18–22], including many membrane transduction systems, and has been implicated in a number of diseases, such as addiction, pain, cancer and psychiatric disorders [23–25]. A 2.5 Å resolution crystal structure of the human sigma_1_ receptor in complex with two chemically divergent ligands, PD144418 and 4-IBP, revealed a trimeric structure with a single transmembrane domain followed by a cupin-like β-barrel domain with the ligand binding site embedded at its center, providing the fundamental basis for understanding its ligand binding, receptor oligomerization and function [26].

**Fig 1:**
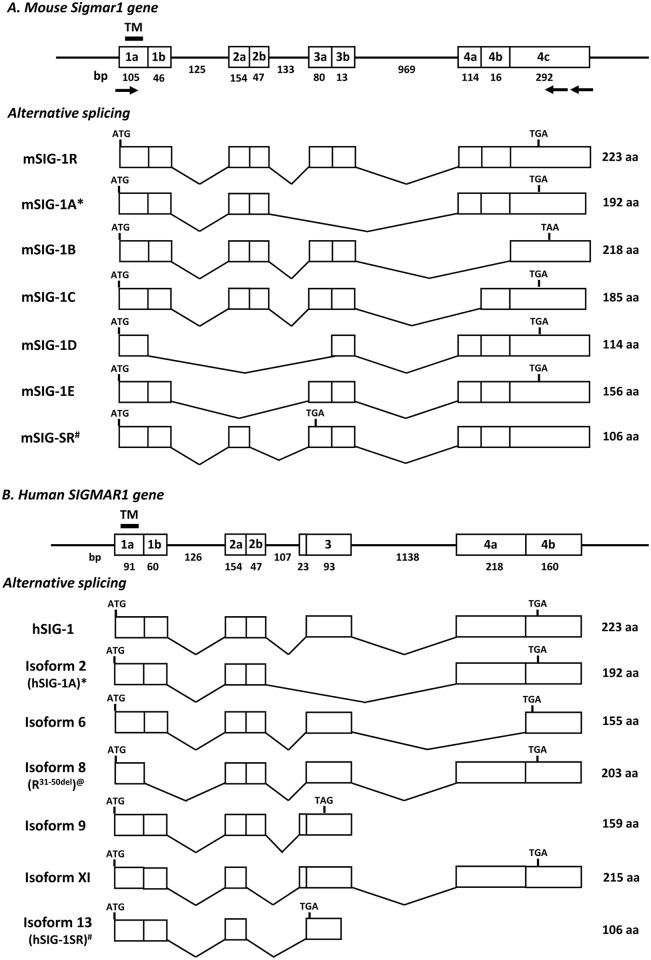
Schematic of the sigma_1_ receptor gene structure and alternative splicing. A). The mouse sigma_1_ receptor gene (Sigmar1). B). The human sigma_1_ receptor gene (SIGMAR1). Exon and introns are showed by boxes and horizontal lines, respectively. The predicted transmembrane domain is encoded by exon 1a and indicated by a short heavy line. Arrows represent primers used in PCR cloning. Translational start (ATG) and termination codons (TGA or TAA or TAG) are shown at indicated exons. The sizes (bp) of exons and interns are shown below the boxes and lines. The number of the predicted amino acids in each variant is listed at right. * & #: similar splice variants in both mouse and human [27, 28]; @: a human variant reported from literature [29].

Soon after the sigma_1_ receptor cDNA were cloned, the chromosomal location and gene structure of sigma_1_ receptor gene (SIGMAR1 or OPRS1) was identified in mouse chromosome 4 [12, 13] and in human chromosome 9 [16]. The exon-intron structures were very similar among the mouse, rat and human SIGMAR1 genes, with all containing four coding exons with similarly sized introns. The first splice variant of SIGMAR1 gene, Sigma R1A, was isolated from the Jurkat human T lymphocyte cell line [27], in which exon 3 was skipped (Fig 1). Subsequently, a mouse splice variant lacking 47 bp of exon 2 was identified (Fig 1) [28]. Recently, a human SIGMAR1 variant with a 20 amino acid (aa) truncation in exon 1 was identified in patients suffering from hereditary distal muscular atrophy (Fig 1) [29]. Here, we describe an additional series of alternatively splice variants of the mouse Sigmar1 gene.

## Materials and methods

### Ethics statement

Prior to the study, all animal studies were reviewed and approved by the Institutional Animal Care and Use Committee (IACUC) (IACUC protocol #: 90-05-010) of the Memorial Sloan-Kettering Cancer Center that is fully accredited by the Association for Assessment and Accreditation of Laboratory Animal Care and in compliance with the Animal Welfare Act, PHS Policy, and other Federal statutes and regulations relating to animals and experiments involving animals.

### Animals and materials

CD-1 and C57BL/6J male mice at 6–8 weeks of age were obtained from Charles River Laboratories and Jackson Laboratory, respectively. [^3^H] (+)-Pentazocine was purchased from PerkinElmer (Boston, MA). HEK293 cells were obtained from ATCC (Manassas, VA). All oligonucleotides were synthesized and purified by Sigma-Aldrich (St. Louis, MO). All other materials were obtained from the sources listed.

### Tissue dissection, RNA extraction and Reverse-Transcription (RT)

Tissues including mouse lung, heart, liver, spleen, intestine, kidney and whole brain were collected immediately after sacrifice. Brain regions, including the prefrontal cortex (PFC), striatum (Str), thalamus (Tha), brainstem (BS), and cerebellum (Cb) were dissected on a mouse Plexiglas brain mold using the atlas of Paxinos and Franklin as a reference. The dissected tissues were immediately homogenized in QiAzol Reagent (Qiagen). Total RNAs were extracted using miRNeasy kit (Qiagen) with DNase I on-column digestion following the manufacture’s protocol. RNAs were quantified using a Qubit 2.0 Fluorometer (Invitrogen), and used in RT reactions with Superscript II (Invitrogen) or VeriScript reverse transcriptase (Affymetrix) and random hexmers. The first-strand cDNA was then used as a template in regular PCR for cloning or in SYBR qPCR for quantification.

### PCR cloning

The first-strand cDNAs from the brain of CD-1 mice were used as a template to isolate Sigmar1 splice variants using Platinum Taq DNA polymerase with a sense primer (pSE1:5’-GTA GGA TCC ATG CCG TGG GCC GCG GG-3’) and an antisense primer (pAN1: 5’-GCA TCT CTG TGT CTC ATT TGC TTC CC-3’; or pAN2: GTT GAA TTC GAG AGA TGG ATG TGG TCC TGC CGC-3’). The PCR fragments with different sizes were excised and purified from an agorase gel using a Gel DNA Recovery Kit (ZYMO Research) and subcloned into a pCRII-TOPO plasmid (Invitrogen). The subcloned PCR fragments were sequenced using appropriate primers.

### Cell culture, plasmid constructs and transient or stable transfection

HEK293 cells (ATCC) were maintained in DME/NEAA-F12 plus high glucose medium supplemented with 10% fetal calf serum at 37°C in a 5% CO_2_/95% air humidified atmosphere. To express the Sigmar1 splice variants in HEK293 cells, the DNA fragments in pCRII-TOPO were subcloned into pcDNA3.1 or pcDNA3.1/Zeo vector (Invitrogen) with appropriate restriction enzyme sites. Carboxyl (C)-terminal HA tagged variant constructs were made using PCR with primers containing an HA tag sequence and subsequently subcloning the PCR fragments into pcDNA3.1 or pcDNA3.1/Zeo vector. The resulting plasmids were transiently transfected into HEK293 cells using Effectene reagent (Qiagen) for binding studies. For dimerization studies, the HA-tagged Sigmar1 variant constructs were transiently co-transfected with the Flag-tagged mMOR-1/pcDNA3.1 construct (mMOR-1/Flag) in HEK293 cells [30]. To examine the effect of Sigmar1 variants on dimerization of mMOR-1 and mSigmar1, equal amounts of mMOR-1/Flag and HA-tagged mSigmar1 (mSIG-1/HA) constructs were transiently co-transfected together with varying amounts of untagged mSigmar1 variants in HEK293 cells. The transfected cells were harvested 48 hours after transfection for immunoprecipitation (IP) studies.

### Immunoprecipitation and western blot analyses

Whole cells from transient transfections were solubilized in lysis buffer A, (phosphate-buffered saline (PBS), pH7.4, CHAPS (3 mM) and a protease inhibitor cocktail (2 μg/ml each leupeptin, pepstatin, aprotinin, and bestatin, and 0.2mM phenylmethylsulfonyl fluoride (PMSF)), and shaken at 4°C for 5 h. The mixture was centrifuged at 13,000g for 15 min at 4°C. The lysate supernatant was incubated with EZview Red Anti-Flag M2 or EZview Red Anti-HA Affinity Gels (Sigma) with shaking overnight at 4°C. After washing with washing buffer (PBS, pH7.4, CHAPS (5 mM)), the affinity gels were used in binding assays (see below) or eluted with 3xFLAG peptide or HA peptide (Sigma) for Western blot. The elutes were mixed with SDS sample buffer containing 0.15 M dithiothreitol (DTT) and heated at 100°C for 10 min. The samples were separated on a 4–20% gradient SDS-PAGE gel and transferred onto PVDF membranes. The membranes were blocked in a blocking solution containing TTBS (10mM Tris—HCl, pH 7.4, 150mM NaCl, and 0.05% Tween 20), 4% nonfat dried milk, and 1% BSA at room temperature for 1 h and incubated with the anti-HA antibody (Santa Cruz) or the anti-FLAG antibody (Sigma) (1:1,1000 dulution) in the blocking solution at 4°C overnight. After washing with TTBS buffer, the membrane was incubated with peroxidase-conjugated goat anti-rabbit or goat-anti-mouse IgG antibody (1:10,000 dilution, JacksonImmuno) in TTBS buffer at room temperature for 1 h. After washing with TTBS buffer, the signals were determined by using ChemiGrow reagents (Proteinsimple, Santa Clara, CA), exposed on Kodak BioMax film, imaged and analyzed on FC8000 Image System (Proteinsimple).

### In vitro transcription coupled with translation

Sigmar_1_ variant constructs in pcDNA3.1 were transcribed and translated in vitro with a TNT-coupled reticulocyte lysate kit (Promega, Madison, WI). Briefly, the plasmids were incubated with T7 RNA polymerase and reticulocyte lysate in the presence of 0.04 mCi of [^35^S]methionine (.1000 Ci/mmol; DuPont-NEN, Boston, MA) at 30°C for 1 h. The translation products were separated by a 4–15% gradient SDS-polyacrylamide gel, and the gel was treated with Amplify (Amersham Life Science), dried, and exposed to Kodak BioMax MR film.

### SYBR green qPCR

The first-strand cDNAs reverse-transcribed from RNAs of various tissues and brain regions were used as templates in SYBR qPCR using Hot Start SYBR Green Master Mix (Affimetrix, Santa Clara, CA) with CFX-96 machine (Bio-Rad). To analyze gene expression levels across multiple tissues more accurately, we used several housekeeping genes, as recommended in the literature [31, 32]. We used five reference genes, including the TATA box binding protein (TBP) and glyceraldehyde 3-phosphate dehydrogenase (G3PDH), succinate dehydrogenase subunit A (SDHA), 18S ribosomal RNA (18S) and β2 microglobulin (B2M). C(t) values from five reference genes were used to calculate normalization factor (NF) following the formula [31]:

$$NF=\;\sqrt[{5}]{C\left(t\right)TBP\times C\left(t\right)G3PDH\times C\left(t\right)SDHA\times C\left(t\right)18S\times C\left(t\right)B2M}$$

Normalized expression (NE) for each variant was calculated using the formula to obtain delta C(t)(ΔC(t)):

ΔC(t) =C(t)varaint −NF

[33]. PCR primers and conditions are listed in S1 Table (see Supplemental Information).

### Receptor binding assays

Membranes were prepared from the transfected HEK293 cells, as previously described [34]. [^3^H](+)-Pentazocine binding and [^3^H]1,3-di-o-tolylguanidine ([^3^H]DTG) binding was performed at 37°C for 150 min in 10 mM potassium phosphate buffer, pH 7.4. [^3^H]DTG binding included unlabeled (+)-Pentazocine (1 μM) to block sigma_1_ binding, leaving only sigma_2_ binding. Specific binding was defined as the difference between total binding and nonspecific binding, defined by haloperidol (1 μM). Protein concentration was determined by the Lowry method using BSA as the standard. Binding to immunoprecipitated receptor was performed as described previously [13]. Briefly, cleared whole cell lysate were immunoprecipitated using EZview Red Anti-HA Affinity Gels in 1.5 ml tubes as described above. Binding on immunoprecipitated affinity gels was performed in 1.5 ml tube as described above except that, instead of filtration., the bound and free ligand were separated by centrifugation, followed by a single wash of the pellet with binding buffer. The pellet was soaked in scintillation fluid overnight and counted in a Scintillation Liquid Analyzer (TRI-CARB 2900TR, PerkinElmer).

### Statistical analysis

Analysis of qPCR data utilized one-way ANOVA with *post hoc* Bonferroni’s multiple comparison test. Data are represented as the mean ± SEM of at least three independent determinations. Statistical significance was set at *p* < 0.05.

## Results

### Molecular cloning of alternatively spliced Sigmar1 variants

**Fig 2:**
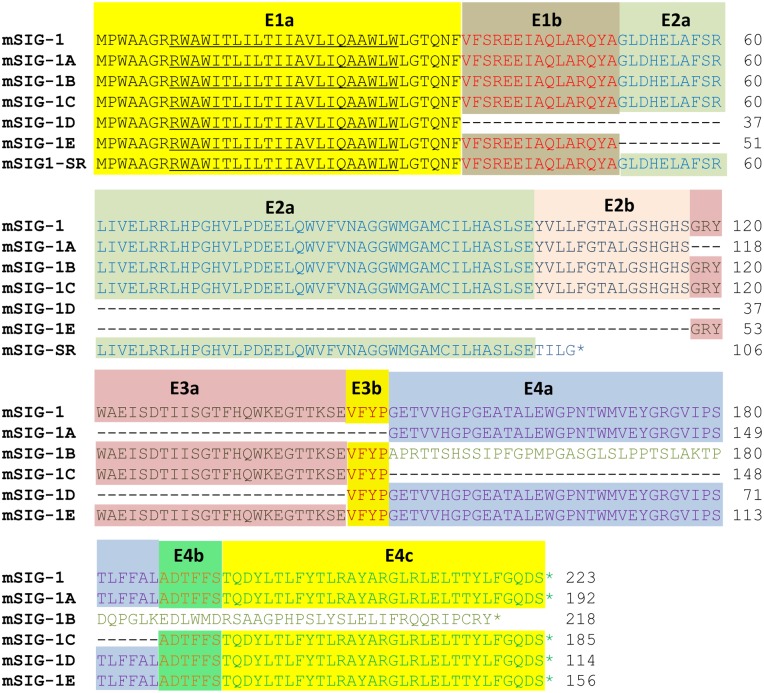
Alignment of the predicted amino acid sequences of the mouse Sigmar1 splice variants. The predicted amino acid sequences were aligned using ALIGN progrom in Vector NTI (Invitrogen). The exon assignment for the predicted amino acids is shown by colored letters and backgrounds. The transmembrane domain is underlined. The number of amino acids is list on the right. *: stop codon. The nucleotide and deduced amino acid sequences of the mouse sigma1 receptor 1a (mSIG-1A), mSIG-1B, mSIG-1C, mSIG-1D and mSIG-1E have been deposited in the GenBank database with Accession numbers: AY390764, AY390765, AY390766, AY390767 and AY390768, respectively.

To isolate the mouse Sigmar1 splice variants, we used a PCR approach with an upstream sense primer in the first exon and a downstream antisense primer in the fourth exon. This led to the identification of five splice variants, mSIG-1A, mSIG-1B, mSIG-1C, mSIG-1D and mSIG-1E (Figs 1 & 2). mSIG-1A and mSIG-1E are exon skipping variants that skip exon 3 and exon 2, respectively. Therefore, the deduced amino acids of mSIG-1A and mSIG-1E are identical to those of the original mSIG-1, except for the truncation of 31 and 67 amino acids encoded by exons 3 and 2, respectively. The mSIG-1A is a homolog of the human Sigma R1A variant isolated earlier [27]. mSIG-1B and mSIG-1C are alternative 3’ splicing variants produced through alternative 3’ acceptor sites in exon 4. Both mSIG-1B and mSIG-1C share the same 148 amino acid sequences as mSIG-1 up to exon 3, but splicing from exon 3 to alternative acceptor sites in exon 4 predicts different C-terminal sequences. Thus, the last 70 amino acids encoded by exon 4c in mSIG-1B are totally different from those in mSIG-1 due to read-frame shifting. Splicing from exon 3 to exon 4b in mSIG-1C leads to truncation of 38 amino acids encoded by exon 4a. mSIG-1D is a splice variant derived from combination of alternative 5’ splicing in exon 1, exon 2 skipping and alternative 3’ splicing in exon 3. So mSIG-1D predicts 114 aa by truncating 109 aa from exons 1b (15 aa), 2 (67 aa) and 3a (27 aa). All of the splice sites were in agreement with the GT/AG rule.

### Expression of mSigmar1 splice variant mRNAs

**Fig 3:**
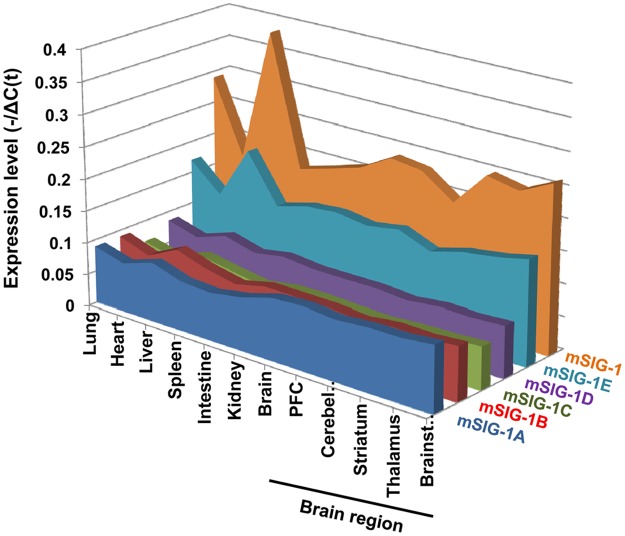
Overall expression levels of the mouse Sigmar1 splice variant mRNAs. Expression levels (-1/ΔC(t)) of the mouse Sigmar1 splice variant mRNAs is plotted across tissues and brain regions using MS excel. ΔC(t) values were determined as: Δ*C*(*t*)*variant* − *Normalized factor (NF)* [33] from at least three independent samples in one experiment. PFC: prefrontal cortex. SYBR green qPCR was performed as described in Materials and Methods. All the primer sequences and qPCR conditions are listed in S1 Table.

**Fig 4:**
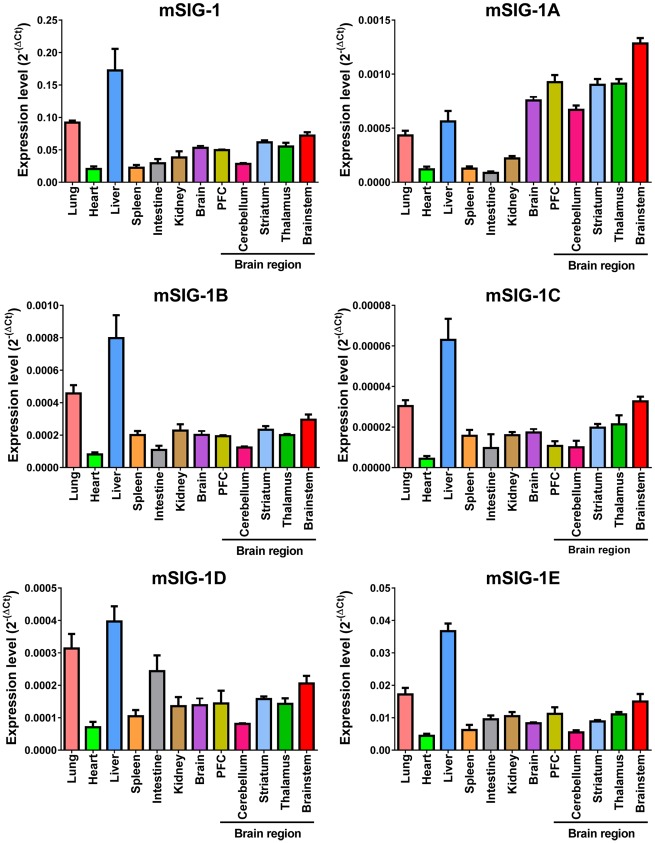
Expression of individual mouse Sigmar1 splice variant mRNAs. Each panel represents one of the mouse Sigmar1 splice variants. Bars represent the mean of 2^-ΔC(t)^ values ± S.E.M. from at least three independent samples in one experiment. ΔC(t) values were determined as: Δ*C*(*t*) = *C*(*t*)*variant* − *Normalized factor (NF)* [33]. PFC: prefrontal cortex. Significant difference was calculated by One-way ANOVA with Tukey’s multiple comparisons test (Prism 6.0). The results of the statistical analysis were listed in S2 Table.

We next examined the expression of the splice variant mRNAs in several tissues and brain regions using SYBR green qPCR. The expression patterns of the splice variants among the tissues and brain regions are shown in Fig 3 based on the values (-/ΔC(t)) of individual variants. The overall expression level of the original sigma_1_ receptor (mSIG-1) was highest in all tissues and brain regions when compared to any of the splice variants, suggesting that mSIG-1 is the predominant mSigmar1 gene transcript. The rank order of the overall expression levels among the splice variants is: mSIG-1E > mSigma-1A > mSIG-1B > mSIG-1D > mSIG-1C (Fig 3). Plotting the expression level (2^-ΔC(t)^) for each variant among the tissues and regions shows the differences quantitatively and provides a visual pattern of tissue or regional expression (Fig 4). For example, the liver showed highest expression for all the variants among the tissues examined, except for mSIG-1A. In contrast, the heart had the lowest expression of all the variants. The overall expression patterns of most variants, including mSIG-1B, mSIG-1C, mSIG-1E, and mSIG-1, were very similar among the tissues. However, we observed a different pattern with mSIG-1A. Its expression levels in brain were higher than the other tissues, including liver, contrasting with the lower levels of the other variants in brain compared to liver. Similarly, the expression of mSIG-1D was relatively higher in the intestine than the other variants. These results suggest tissue-specific alternative splicing of the mSigmar1 gene.

Several mSigmar1 splice variants showed brain region-specific expression. For example, mSIG-1A expression was highest in the brainstem with low levels in the cerebellum, striatum and thalamus. Similarly, the brainstem had higher expression of mSIG-1C than the prefrontal cortex (PFC) and cerebellum.

### Characterization of mSigmar1 splices variants

**Fig 5:**
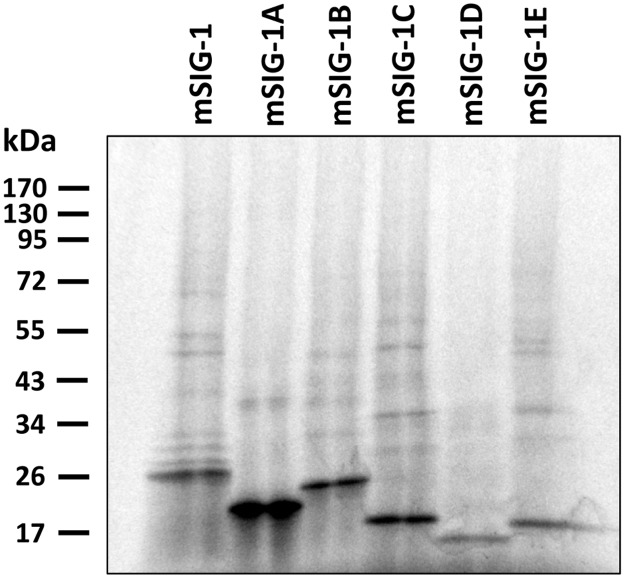
In vitro transcription coupled with translation. In vitro transcription coupled with translation was performed using a TNT-coupled reticulocyte lysate kit (Promega) as described in Materials and Methods. The translation products were separated by a 4–15% gradient SDS-polyacrylamide gel, and the gel was treated with Amplify (Amersham Life Science), dried, and exposed to Kodak BioMax MR film. The predicted molecular weights of mSIG-1, mSIG-1A, mSIG-1B, mSIG-1C, mSIG-1D and mSIG-1E are 25.3, 21.6, 24.5, 21.2, 13.0 and 17.9 kDa, respectively. The representative gel from two experiments was shown.

*In vitro* transcription coupled translation using cDNA constructs in pcDNA3 vectors revealed molecular weights for all the splice variants corresponding to their predicted sequences (Fig 5). We then transiently transfected HEK293 cells with the pcDNA3 constructs and examined [^3^H]-(+)pentazocine and [^3^H]DTG binding for sigma_1_ and sigma_2_ binding sites, respectively. We observed a robust increase of [^3^H](+)-pentazocine binding in HEK293 cells transfected with the original mSIG-1 over control HEK293 cells transfected with pcDNA3 vector (S1 Fig), consistent with our previous observation [13]. There was no significant [^3^H](+)-pentazocine binding in HEK293 cells transfected with the other mouse variants when compared with control transfected HEK293 cells. [^3^H]DTG binding was carried out in the presence of unlabeled (+)pentazocine, providing a measure of only sigma_2_. No significant increase in [^3^H]DTG binding was observed in any of the transfected cells over control cells, including mSIG-1, confirming that mSIG-1 corresponds to the classical sigma_1_ receptor [^3^H]DTG binding site and illustrating the far lower levels of sigma_2_ binding in HEK293 cells than sigma_1_ sites.

The failure of any variants other than mSIG-1 to increase [^3^H](+)pentazocine binding above endogenous levels implied that the variants did not bind [^3^H](+)pentazocine. To avoid the influence of endogenous sigma_1_ receptor expression on binding, we transfected HEK293 cells with the HA-tagged constructs, which allowed the immunoprecipitation of only the transfected receptor, as previously described [13]. Immunoprecipitated HA-tagged mSIG-1 showed a robust [^3^H](+)-pentazocine binding (S2 Fig). Again, we saw no specific [^3^H](+)-pentazocine binding with any of the other variants, suggesting their inability to bind traditional sigma_1_ ligands.

### Physical associations of mSigmar1 splice variants with mu opioid receptor

**Fig 6:**
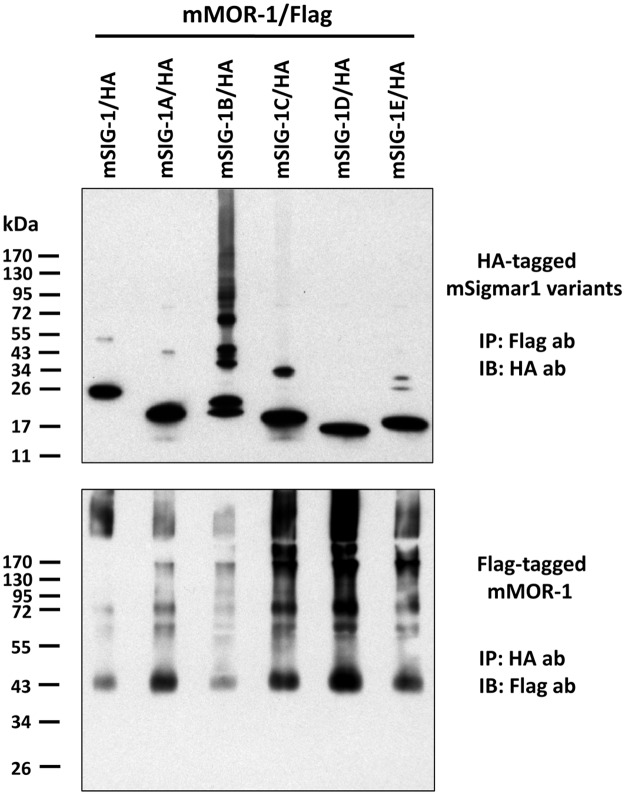
Heterodimerization of mMOR-1 and mSigmar1 splice variants. Flag-tagged mMOR-1 and HA-tagged mSigmar1 variants were transiently co-transfected into HEK293 cells. Co-IP was performed as described in Materials and Methods. Briefly, cleared whole cell lysate was incubated with EZview Red Anti-Flag or anti-HA Affinity gels overnight. After washing, the tagged proteins on the affinity gels were eluted with 3xFlag or HA peptide, and the elutes were analyzed in Western blot. Top panel: Immunoblot (IB) with anti-HA antibody using elutes from immunoprecipitation (IP) with EZview Red Anti-Flag Affinity Gel. Bottom panel: IB with anti-Flag antibody using elutes from IP with EZview Red Anti-HA Affinity Gel. The representative blots from two independent experiments were shown.

The original mSIG-1 physically associates with the mouse mu opioid receptor variant mMOR-1, thereby modulating mu opioid receptor function [19]. We then examined whether the mSigmar1 splice variants physically associated with mMOR-1 in transient co-transfections using HEK293 cells with HA-tagged mSigmar1 variants and Flag-tagged mMOR-1. Imunoprecipitating (IP) the Flag-tagged mMOR-1 with a Flag antibody followed by immunoblotting (IB) with a HA anbody visualized a major band of each HA-tagged splice variants consistent with its predicted molecular weight (Fig 6, Top). A doublet band was seen in mSigmar-1B, possibly due to degradation. We also observed minor bands with molecular weights approximately twice that of the major band for all the splice variants except for mSigmar-1D, suggesting dimerization of these variants under these conditions or association with an unknown protein. Interestingly, we detected multiple forms of mSigmar-1B with even higher weights. These bands may represent oligomers of mSigmar-1B, as suggested by the trimeric crystal structure, or associations with other proteins, possibly the Flag-tagged mMOR-1. It is interesting that we did not observe bands 3-fold higher with the other variants, as might have been expected based upon the trimeric structure seen in the crystal structure [26].

In the inverse experiment in which we pulled down HA-tagged splice variants and immunoblotted for Flag-tagged mMOR-1, Flag-tagged mMOR-1 was co-immunoprecipitated by all the splice variants (Fig 6, Bottom). It is interesting that the most intense band corresponds to an immature, non-glycosylated form of MOR-1, consistent with the established localization of sigma_1_ receptors in the endoplasmic reticulum and its chaperone activity. In addition to bands corresponding to a mature receptor at approximately 75 kDa, several other bands of higher molecular weight were observed. The identity of these is not known, but the possibility of homooligomerization or other associated proteins must be considered. The specificity of the physical association was validated in control studies in which we examined membranes from either a transfection with only a single tagged construct or a mixture of membranes from cells singly transfected HA-tagged variants with the Flag-tagged mMOR-1. In all cases, we saw no co-precipitation in these control conditions (S3 Fig), indicating that the physical association was not an artifact of the solubilization procedure. Together, these results demonstrate that all mSigmar1 splice variants can physically associate with the mu receptor variant mMOR-1 when co-expressed in HEK293 cells.

### Effects of mSigmar1 splice variants on physical association of the original mSIG-1 with mMOR-1

**Fig 7:**
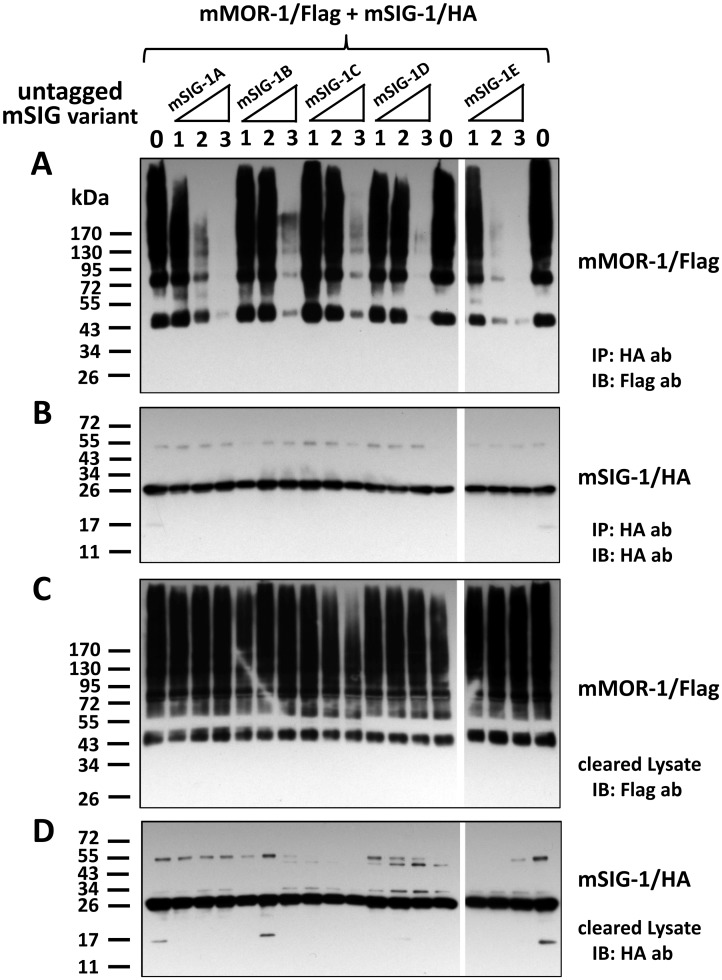
Effects of mSigmar1 splice variants on physical association of the original mSIG-1 with mMOR-1. A). Western blot of HA antibody-precipitated elutes with Flag antibody (ab). Equal amounts of mSIG-1/HA (1.2 μg) and mMOR-1/Flag (1.2 μg) were co-transfected into a 100 mm plate of HEK293 cells, together with each untagged mSigmar1 variant at four different amounts (0 μg, 1.2 μg, 2.4 μg or 3.6 μg, labeled as 0, 1, 2, 3, respectively), by using Effectene (Qiagen) as described in Materials and Methods. Appropriate amount of pcDNA3 vector DNA was added to maintain the total amount of DNA in each transfection as 6 μg. Whole cells were solubilized 48 hr after transfection, and cleared lysate by centrifugation was then used in immunoprecipitation with EZview HA affinity gel. Eluted proteins by Flag peptides were separated by SDS-PAGE and transferred into a PVDF membrane, which was then immunobloted with an anti-Flag antibody, as described in Materials and Methods. The representative blot from two independent experiments was shown. IP: immunoprecipitation; IB: immunoblot. B). Western blot of HA-antibody-precipitated fraction with EZview HA affinity gel. The same elutes were analyzed using an anti-HA antibody in immunobloting. C) & D). Western blot of cleared lysate with anti-Flag antibody or anti-HA antibody. Cleared lysate samples before IP were analyzed using anti-Flag antibody (C) or an anti-HA antibody (D) in immunobloting.

To examine whether mSigmar1 splice variants can have a dominant negative role in dimerization of the original mSIG-1 with mMOR-1, we performed a competition-Co-IP study in which the dimerization of HA-tagged mSIG-1 with Flag-tagged mMOR-1 was competed with increasing amounts of individual non-tagged mSigmar1 splice variants in co-transfected HEK293 cells. In the absence of non-tagged mSigmar1 variants, mMOR-1/Flag was co-immunoprecipitated with mSIG/HA using HA affinity gel (Fig 7A), consistent with our current (Fig 6) and earlier observations [19]. Increasing the levesl of the other mSigmar1 variants lowered co-immunoprecipitated mMOR-1/Flag in a dose-dependent manner (Fig 7A). Similar amounts of mSIG-1/HA were co-immunoprecipitated (Fig 7B), indicating that the efficiency of IP was similar among the samples. At the lowest dose (mSigmar1 variant:mSIG-1/HA = 1:1), there were limited effects on competing the dimerization for all mSigmar1 variants, except for mSIG-1E. When the dose was increased by two-fold (mSigmar1 variant:mSIG-1/HA = 2:1), we observed a great reduction of co-immunoprecipitated mMOR-1/Flag with mSIG-1E and mSIG-1A, while a smaller reduction was seen with mSIG-1C and mSIG-1D. At the highest dose used (mSigmar1 variant:mSIG-1/HA = 3:1), mMOR-1Flag was almost totally competed off with all the mSigmar1 variants. The competing effects of mSIG-1E, mSIG-1A and mSIG-1D were most effective. The expression levels of transfected mMOR-1/Flag and mSIG-1/HA among the samples were relatively similar as indicated by immunoblots using lysate (Fig 7C & 7D). These results suggest that all mSigmar1 splice variants have a dominant negative function in their ability to inhibit dimerization of mSIG-1/HA with mMOR-1/Flag when overexpressed in HEK293 cells.

## Discussion

Alternative pre-mRNA splicing is one of the major mechanisms creating transcriptomic and proteomic diversity. Over 90% of human genes undergo alternative splicing [35, 36]. The current study further reveals extensive alternative splicing of the mouse Sigmar1 gene. Similar alternative splicing has been observed in the human SIGMAR1 gene based on the molecular cloning or genome sequence prediction (Fig 7). The human Sigma R1A [27] is homologous to mSIG-1A. A human isoform 6 has a similar splicing pattern as the mouse mSIG-1B and mSIG-1C. The hSIG-1SR (isoform 13) has identical splicing as the mouse mSIG-1SR [28]. Several other human SIGMAR1 variants are also derived from alternative 3’ or 5’ splicing. All these variants create a series of truncated sigma_1_ receptors at protein level.

The mSigmar1 splice variant mRNAs are expressed in a number of tissues and brain regions in C57BL/6J mice, assessed with SYBR green qPCR. We used five housekeeping genes for normalizing the expression of the variants to minimize potential biases. Although not ideal, it provides an estimation of relative expression levels across tissues under this quantification method. The abundance of the mSigmar1 variant mRNAs covers a wide range. Similar expression patterns in various tissues and brain regions among most of the mSigmar1 variants, including the original mSIG-1, suggest that they share conserved alternative splicing mechanisms. However, the different expression pattern of mSIG-1A and mSIG-1D reveals its brain-specific and intestine-specific alternative splicing, respectively. It will be interesting to investigate their expressions in more tissues and brain regions in the future beyond the limited ones examined in this study.

The truncated mSigmar1 variants provide native deletion mutants for exploring the functional motifs of the sigma_1_ receptor. mSIG-1A has a deletion of 31 aa encoded by exon 3, while mSIG-1E lost 67 aa encoded by exon 2. mSIG-1B has a truncation of 75 aa encoded by the 2^nd^ reading-frame of exons 4a/4b/4c that is replaced by a new stretch of 70 aa encoded by the 3^rd^ reading-frame of exon 4c. Loss of [^3^H](+)-pentazocine binding in these splice variants suggest that all the truncated regions or sequences influence the binding pocket. Early mutagenesis studies showed that various single point substitutions at different positions reduced or abolished sigma_1_ ligand binding [37, 38], suggesting involvement of these residues are important for ligand binding. Interestingly, these mutant positions are located in all the truncated exons, except for exon 1. They include Ser99 (exon 2), Try103 (exon 2), Asp126 (exon 3) and Glu172 (exon 4). Our results are consistent with those from the early studies. The recent sigma_1_ receptor crystal structure revealed that the ligand binding site is at the center of a cupin-like β-barrel structure [26], which is encoded by exons 2/3/4. Therefore, it is not surprising that we observed complete loss of [^3^H](+)-pentazocine binding when the entire corresponding exon was truncated in these splice variants.

Sigma_1_ receptors physically associate with a mu opioid receptor variant (MOR-1) in transfected cells [19]. However, we know little about which motif or sequences are involved with these interactions. Truncated variants at various parts of the receptor offer useful tools for mapping the sequence. Co-immunoprecipitation studies clearly demonstrate that all the truncated variants physically interact with mMOR-1, suggesting that the truncated sequences do not involve the interaction. The only common sequences shared among all the splice variants include the first 35 aa that are encoded by exon 1a and contained in the transmembrane domain (Fig 2). Thus, these results suggest that the 35 aa sequences may involve interaction with mMOR-1. Based on the crystal structure, the transmembrane domain within the 35 aa sequences is outward from the trimeric structure [26], favoring its interaction with other proteins and supporting our hypothesis.

The functions of these mSigmar1 splice variants remain unclear. Although these truncated splice variants are unable to bind the sigma_1_ ligand, they maintain the ability to physically associate with mMOR-1, raising the possibility that they still may be functionally important, perhaps with a dominant negative function. This hypothesis is supported by the observation that all mSigmar1 splice variants were able to disrupt the dimerization of mSIG-1/HA with mMOR-1/Flag when they were overexpressed. Our qPCR data showed that all mSigmar1 splice variant mRNAs were expressed at levels lower than the expression of the original mSIG-1 mRNA, raising questions regarding whether they can dissociate the dimerization of mSIG-1 with mMOR-1 under the physiological or pathological conditions. Nevertheless, these results provide significant insights into the function of mSigmar1 splice variants. Shioda et al. reported that a mouse sigma splice variant (SR) served as a dominant negative form to promote mitochondrial energy depletion and apoptosis by blocking C-terminal chaperone activity or interfering with IP_3_R/sigma_1_ receptor interaction under endoplasmic reticulum stress [28]. It will be interesting to further explore the functional consequence of such a dissociation of mSIG-1 with mMOR-1 by mSigmar1 splice variants in mu opioid receptor function and signaling in the future. While these studies focused upon mu opioid receptors, sigma_1_ receptors have many interactions with a wide range of other proteins. Thus, it remains possible that these variants may provide some specificity among these various protein associations.

In conclusion, we have identified five mouse Sigmar1 splice variants, all of them generated from exon skipping or alternative 3’ or 5’ splicing, producing the truncated sigma_1_ receptors. Several variants showed tissue-specific or region-specific expression at mRNA level. All the truncated variant receptors lost [^3^H](+)-pentazocine binding, but maintained their ability to physically associate with the mu opioid receptor, and functioned as a dominant negative form by disrupting the physical association of the original sigma_1_ receptor with mMOR-1 when overexpressed, providing useful insights into their function.

## Supporting information

S1 Fig [^3^H](+)-Pentazocine and [^3^H]DTG binding on membranes from HEK293 cells transiently transfected with the mouse Sigmar1 variants. [^3^H](+)-Pentazocine and [^3^H]DTG binding were performed for sigma_1_ and sigma_2_ binding, respectively, as described in Materials and Methods. Briefly, membranes were isolated from HEK293 cells transiently transfected indicated Sigmar1 variant constructs or pcDNA3 vector, and used in [^3^H](+)-Pentazocine (~ 1 nM) and [^3^H]DTG binding (~1 nM) in the absence or presence of 1 μM haloperidol to define specific binding. In [^3^H]DTG binding, 1 μM nonradioisotope-labeled (+)-Pentazocine was also included to block sigma_1_ binding. The results were from two independent samples in one experiment.
(PDF)

S2 Fig [^3^H](+)-Pentazocine binding on immunoprecipitated beads. [^3^H](+)-Pentazocine binding on immunoprecipitated gels was performed as described in Materials and Methods. Briefly, cleared whole cell lysate were immunoprecipitated using EZview Red Anti-HA Affinity Gels in 1.5 ml tube. After washing, the Affinity Gels were used in [^3^H](+)-Pentazocine binding. Specific binding was defined by differences between the absence and presence of 1 μM haloperidol. The bound and free radioisotope-labeled ligand were separated by centrifugation, and followed by a single wash of the pellet with binding buffer. The pellet was soaked in scintillation fluid overnight and counted in a Scintillation Liquid Analyzer. The results were from two independent samples in one experiment.
(PDF)

S3 Fig Co-immunoprecipitation study. Top panel: Co-IP study. Co-IP was performed using the membranes from HEK293 cells transfected with single tagged constructs (Lines 1–7) or the same membranes from Line 2–7 mixed with membranes from HEK293 cells transfected with Flag-tagged mMOR-1 construct (Lines 8–13). EZview Red Anti-HA Affinity Gel was used in immunoprecipitation (IP). HA peptide-eluted proteins were used in immunoblot (IB) with anti-Flag antibody. Line 14: lysate from HEK293 cells transfected with mMOR-1/Flag without IP. Bottom panel: The same eluted samples were used in IB with anti-HA antibody. The results were from one experiment.
(PDF)

S1 Table SYBR green qPCR primers and conditions. (XLSX)

S2 Table One-way ANOVA analysis of SYBR green qPCR. (XLSX)
