# Supplementary material for: Isolation and characterization of alternatively spliced variants of the mouse sigma1 receptor gene, Sigmar1
Source: PLoS One. 2017 Mar 28;12(3):e0174694. doi: 10.1371/journal.pone.0174694 (PMC5370144; doi:10.1371/journal.pone.0174694)
Supplement: S1 Fig — [3H](+)-Pentazocine and [3H]DTG binding were performed for sigma1 and sigma2 binding, respectively, as described in Materials and Methods. Briefly, membranes were isolated from HEK293 cells transiently transfected indicated Sigmar1 variant constructs or pcDNA3 vector, and used in [3H](+)-Pentazocine (~ 1 nM) and [3H]DTG binding (~1 nM) in the absence or presence of 1 μM haloperidol to define specific binding. In [3H]DTG binding, 1 μM nonradioisotope-labeled (+)-Pentazocine was also included to block sigma1 binding. The results were from two independent samples in one experiment. (PDF) [file pone.0174694.s001.pdf]

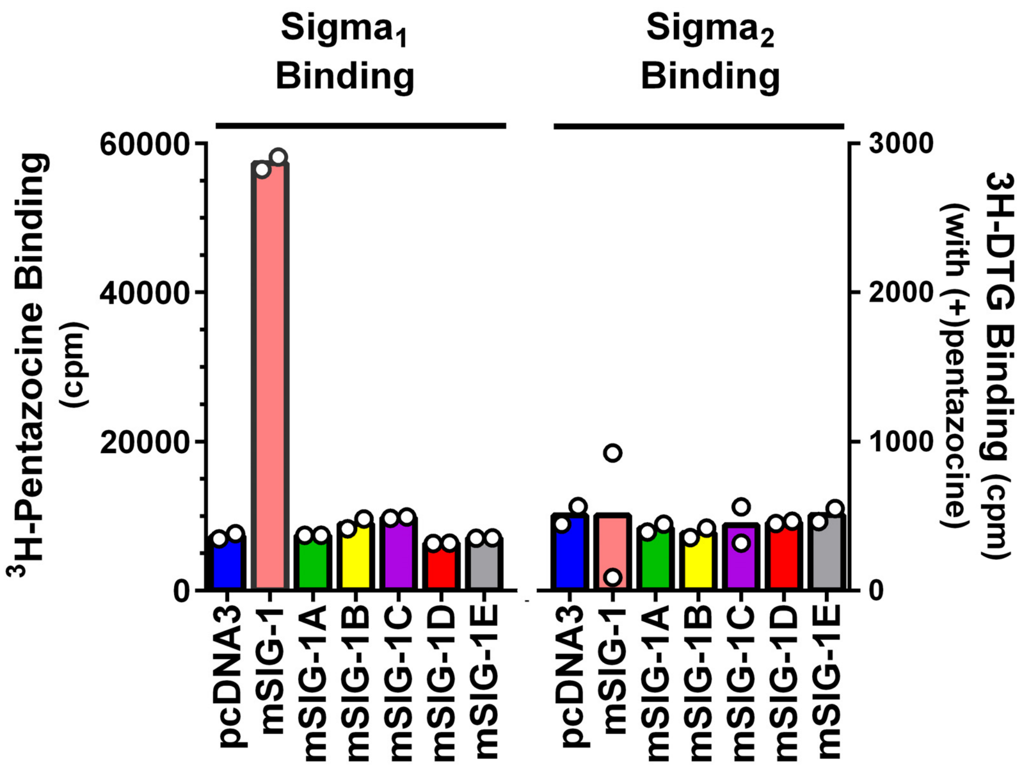

S1 Figure. [^{3}H](+)-Pentazocine and [^{3}H]DTG binding on membranes from HEK293 cells transiently transfected with the mouse Sigmar1 variants.

[^{3}H](+)-Pentazocine and [^{3}H]DTG binding were performed for sigma$_1$ and sigma$_2$ binding, respectively, as described in Materials and Methods . Briefly, membranes were isolated from HEK293 cells transiently transfected indicated Sigmar1 variant constructs or pcDNA3 vector, and used in [^{3}H](+)-Pentazocine (~ 1 nM) and [^{3}H]DTG binding (~1 nM) in the absence or presence of 1 μM haloperidol to define specific binding. In [^{3}H]DTG binding, 1 μM nonradioisotope-labeled (+)-Pentazocine was also included to block sigma$_1$ binding. The results were from two independent samples in one experiment.
