# Supplementary material for: Isolation and characterization of alternatively spliced variants of the mouse sigma1 receptor gene, Sigmar1
Source: PLoS One. 2017 Mar 28;12(3):e0174694. doi: 10.1371/journal.pone.0174694 (PMC5370144; doi:10.1371/journal.pone.0174694)
Supplement: S2 Fig — [3H](+)-Pentazocine binding on immunoprecipitated gels was performed as described in Materials and Methods. Briefly, cleared whole cell lysate were immunoprecipitated using EZview Red Anti-HA Affinity Gels in 1.5 ml tube. After washing, the Affinity Gels were used in [3H](+)-Pentazocine binding. Specific binding was defined by differences between the absence and presence of 1 μM haloperidol. The bound and free radioisotope-labeled ligand were separated by centrifugation, and followed by a single wash of the pellet with binding buffer. The pellet was soaked in scintillation fluid overnight and counted in a Scintillation Liquid Analyzer. The results were from two independent samples in one experiment. (PDF) [file pone.0174694.s002.pdf]

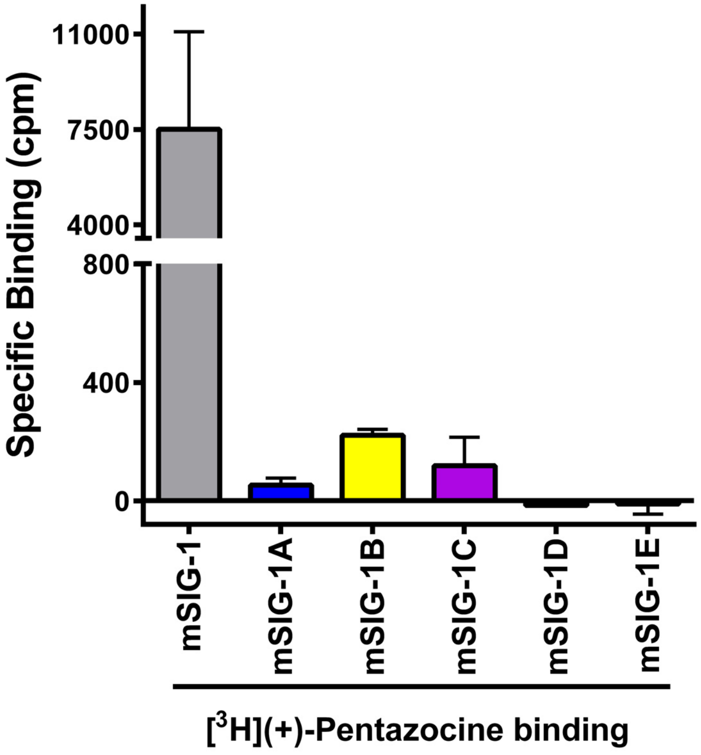

S2 Figure. [^{3}H](+)-Pentazocine binding on immunoprecipitated beads.

[^{3}H](+)-Pentazocine binding on immunoprecipitated gels was performed as described in Materials and Methods. Briefly, cleared whole cell lysate were immunoprecipitated using EZview Red Anti-HA Affinity Gels in 1.5 ml tube. After washing, the Affinity Gels were used in [^{3}H](+)-Pentazocine binding. Specific binding was defined by differences between the absence and presence of 1 μM haloperidol. The bound and free radioisotope-labeled ligand were separated by centrifugation, and followed by a single wash of the pellet with binding buffer. The pellet was soaked in scintillation fluid overnight and counted in a Scintillation Liquid Analyzer. The results were from two independent samples in one experiment.
