# Supplementary material for: Isolation and characterization of alternatively spliced variants of the mouse sigma1 receptor gene, Sigmar1
Source: PLoS One. 2017 Mar 28;12(3):e0174694. doi: 10.1371/journal.pone.0174694 (PMC5370144; doi:10.1371/journal.pone.0174694)
Supplement: S3 Fig — Top panel: Co-IP study. Co-IP was performed using the membranes from HEK293 cells transfected with single tagged constructs (Lines 1–7) or the same membranes from Line 2–7 mixed with membranes from HEK293 cells transfected with Flag-tagged mMOR-1 construct (Lines 8–13). EZview Red Anti-HA Affinity Gel was used in immunoprecipitation (IP). HA peptide-eluted proteins were used in immunoblot (IB) with anti-Flag antibody. Line 14: lysate from HEK293 cells transfected with mMOR-1/Flag without IP. Bottom panel: The same eluted samples were used in IB with anti-HA antibody. The results were from one experiment. (PDF) [file pone.0174694.s003.pdf]

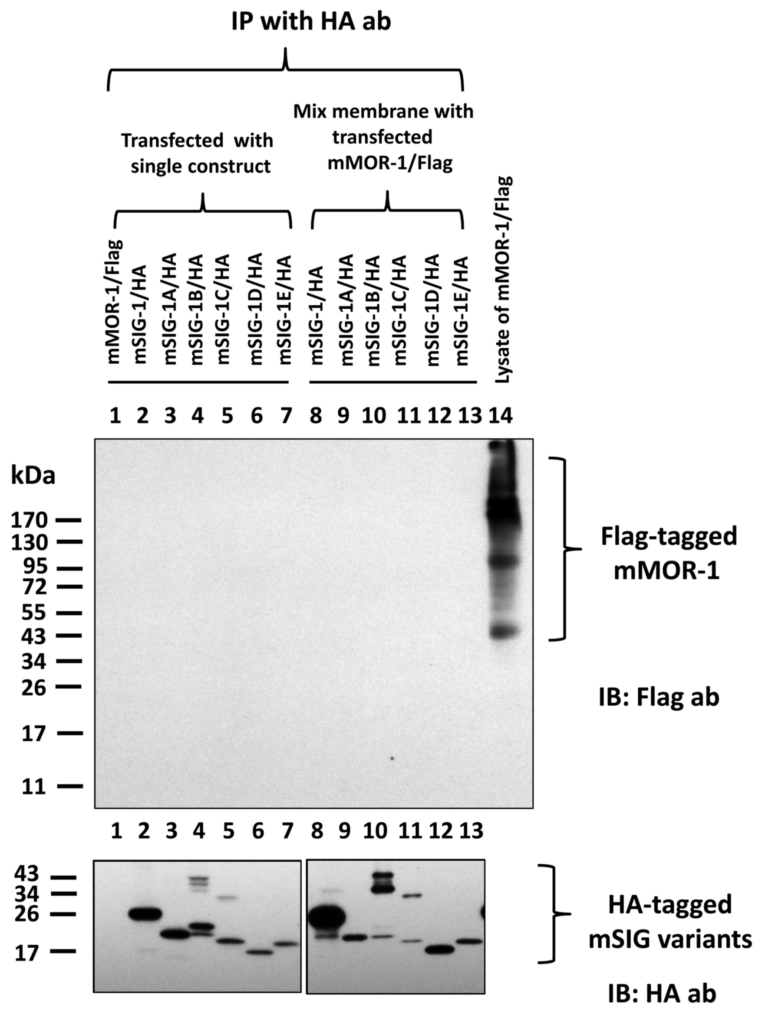

S3 Figure. Co-immunoprecipitation study.

Top panel: Co-IP study. Co-IP was performed using the membranes from HEK293 cells transfected with single tagged constructs (Lines 1 – 7) or the same membranes from Line 2 – 7 mixed with membranes from HEK293 cells transfected with Flag-tagged mMOR-1 construct (Lines 8 – 13). EZview Red Anti-HA Affinity Gel was used in immunoprecipitation (IP). HA peptide-eluted proteins were used in immunoblot (IB) with anti-Flag antibody. Line 14: lysate from HEK293 cells transfected with mMOR-1/Flag without IP. Bottom panel: The same eluted samples were used in IB with anti-HA antibody. The results were from one experiment.
